# Monitoring of physical health in children and young people on antipsychotics in a community CAMHS setting

**DOI:** 10.1192/j.eurpsy.2026.11122

**Published:** 2026-07-31

**Authors:** S. Shafique, B. Poornamodan, L. Rhys

**Affiliations:** 1 North Staffordshire Combined Healthcare NHS Trust, Stoke on Trent; 2 Keele University School of Medicine, Newcastle, United Kingdom

## Abstract

**Introduction:**

Children and young people are particularly vulnerable to the adverse side effects of antipsychotics, including rapid weight gain and metabolic disturbances. Regular monitoring allows antipsychotics to be adjusted so that side effects are minimised and physical health interventions offered if needed.

**Objectives:**

To assess whether NICE guidelines are being adhered to in monitoring physical health in young people who are prescribed antipsychotics. An audit was undertaken for this purpose.

**Methods:**

Children who were prescribed antipsychotics were identified after discussion with the prescribers on the team. Clinical notes were also reviewed in the period from May 2024- April 2025. Data was collected on a) Proportion of young people with a newly prescribed antipsychotic medication who have a record of baseline blood tests, physical health investigations and a record of side-effect monitoring within 12 weeks after starting treatment, and b) Proportion of young people with prescribed antipsychotic medication with a record of regular monitoring of blood tests, physical health parameters and side-effect monitoring within the last 6 months.

**Results:**

There were 7 patients who were on antipsychotics in our catchment area. None of them were started on medication in the audit period so an audit of baseline monitoring could not be done. Monitoring of young people who are on regular antipsychotics was variable. With regards to physical measurements, most (n=5) had a height and weight measurement done but this was not plotted on a growth chart as advised by the NICE guidelines. Waist and hip circumference was not measured at all. Similarly, blood tests monitoring blood glucose, lipids and prolactin were only done yearly whilst none of them were done 6 monthly as recommended. Monitoring of other parameters like efficacy (n=0), adherence (n=4), side effects (n=3) and movement disorders (n=0) were variable.

**Conclusions:**

The national NICE guidelines were not being strictly adhered to in monitoring the children and young people who are prescribed antipsychotics. An action plan was prepared to disseminate information regarding the guidelines and ensure monitoring is being completed appropriately.

**Disclosure of Interest:**

None Declared

